# Supplementary material for: Adoption of the voluntary conflict of interest statement on PubMed
Source: PLoS One. 2024 Oct 30;19(10):e0308782. doi: 10.1371/journal.pone.0308782 (PMC11524491; doi:10.1371/journal.pone.0308782)
Supplement: Attachment — Submitted filename: Response to Reviewers.docx [file pone.0308782.s001.docx]

***Comments from the Editor: I would suggest the authors provide a flow chart showing how study samples were selected/obtained.***

Response: Thank you for the suggestion. We have added a flow chart to the manuscript detailing how the articles included in our study were selected and which analyses were performed on which subsets of the full dataset.

***Reviewers 1 and 2 answer all of the 5 standard questions affirmatively except Reviewer 1 states that we have not made the data underlying the manuscript fully available.***

Response: The data have been submitted to Dryad. The reviewer URL is here: <https://datadryad.org/stash/share/zZLdgW-pVjwxzhH6Y07sk-eUee_Egzrq_Q3dwK0pGDc>. The unique digital object identifier for the data is here: doi:10.5061/dryad.h70rxwds8.

***Reviewer #1: Stephanie and colleagues have done a very good job in this area of rare interest. They have deeply searched, sieved, extracted, collated and synchronized the facts from the ocean of available information/journals. It is worth the effort put into it. The manuscript is generally well written and thus not difficult to follow through. They have also identified the key limitations which is of great importance for further/future studies It is highly commendable.***

Response: Thank you for these comments.

***1. Is it possible for NLM or associated bodies or other groups of people with keen interest to alleviate this identified problem through development and use of applicable software/s?***

Response: Thank you for your question. As mentioned in the discussion, the JATSforReuse group, which is a voluntary collaboration between publishers and NLM, has developed and published recommendations for tagging conflict-of-interest statements to standardize how the content is presented on archiving platforms, including PubMed. However, we are unaware to what extent it is used by the journals.

***2. Can there be any application of artificial intelligence (AI) in solving this identified problem either soon or much later in the future?***

Response: Another good question. While we can see AI being useful in facilitating use of the statement in the future, this question is beyond the scope of our expertise and this article.

***Reviewer #2: I think the statistical analysis of the manuscript was appropriately and rigorously performed and data aligned with the conclusion. Data underlying the findings were made available. The author presented the manuscript in an intelligible way and the written English appropriate.***

Response: Thank you for your observations. The reviewer is correct that the data are available in Dryad.
